# Ulipristal acetate for use in medical management of early pregnancy loss: a pilot feasibility study

**DOI:** 10.1186/s12884-026-09485-4

**Published:** 2026-07-02

**Authors:** Jill M Hagey, Jennifer H Tang, Jessica E Morse, Amy G Bryant

**Affiliations:** 1 Division of Women’s Community and Population Health, Duke Department of Obstetrics and Gynecology, Duke University, DUMC 3084, 236 Baker House 200 Trent Drive, 2301 Erwin Road, Durham, NC 27710 USA; 2 Division of Complex Family Planning, Department of Obstetrics and Gynecology, University of North Carolina, 101 Manning Drive, Chapel Hill, NC 27514 USA

**Keywords:** Early pregnancy loss, Miscarriage, Ulipristal acetate (UPA), Mifepristone, Medical management of miscarriage, Pilot study, Feasibility

## Abstract

**Abstract:**

Mifepristone availability for medical management of early pregnancy loss (EPL) is limited in some settings. We report a single site, single arm pilot of ulipristal acetate for EPL management as an alternative to mifepristone. While recruitment was challenging, all participants adhered to study protocol and passed their pregnancy without additional intervention.

**Trial registration:**

ClinicalTrials.gov (ID NCT05216952), registered January 19, 2022, https//clinicaltrials.gov/study/NCT05216952.

## Objective

Early pregnancy loss (EPL) affects approximately 10% of all women [1]. Pretreatment with mifepristone, a selective progesterone receptor modulator, prior to misoprostol increases effectiveness of medical management of EPL and decreases the need for subsequent surgical management [2]. While only 71% of individuals have resolution of EPL by three days using misoprostol alone, initial studies reported complete pregnancy expulsion in 84% of patients pre-treated with mifepristone; suggesting that the addition of mifepristone allows for more patient-centered medication management of EPL, particularly for those looking to avoid a subsequent procedure [2, 3]. However, mifepristone is subject to regulations based on the US Food and Drug Administration (FDA) Risk Evaluation and Mitigation Strategy (REMS) despite extensive safety data [4].

Ulipristal acetate (UPA) is another selective progesterone receptor modulator that allows for similar priming of the endometrium and sensitization of the myometrium to prostaglandins to potentially improve effectiveness of misoprostol in medical management of EPL [5]. UPA is not subject to the FDA REMS restrictions and is available as a prescription medication for emergency contraception using a one-time dose of 30mg [6]. New data also suggests that UPA may be used in conjunction with misoprostol for pregnancy termination through 9 weeks gestation with high success [7]. Our goal was to identify the feasibility of using UPA as an adjunct to misoprostol for medical management of EPL in place of mifepristone.

## Study design

We report a Phase 2 pilot study of UPA for medical management of EPL at a single academic medical center in Chapel Hill, North Carolina. The study was approved by the University of North Carolina Office of Human Research Ethics (21-2315) and the FDA Investigational New Drug Application and registered prospectively through ClinicalTrials.gov (NCT05216952). This study adhered to the ethical principles outlined in the Declaration of Helsinki.

Patients were eligible for the study if they were over 18 years of age, English- or Spanish-speaking, and diagnosed with a first trimester pregnancy loss between 5- and 12-weeks’ gestation or an anembryonic gestation by ultrasound criteria [8]. After providing written informed consent, participants completed a demographic questionnaire and received baseline laboratory studies (complete blood count, Rhesus blood testing, liver function testing). Participants received the study intervention of 90 mg UPA and were instructed to self-administer 800mcg misoprostol vaginally 6 to 18 hours after UPA. This study utilized UPA 90 mg to most closely match the effects of mifepristone 200 mg based on endometrial histology [9]. Timing of misoprostol administration following UPA is based on a post-hoc analysis of misoprostol administration following mifepristone for EPL indicating the highest likelihood of success when misoprostol was administered 7 to 20 hours after mifepristone [10]. Pharmacokinetic data suggest that peak concentrations of UPA and its active metabolite are 0.9 hours and 1 hour respectively, compared to mifepristone that reaches peak concentrations after 90 minutes; thus we propose earlier times for UPA administration when compared with those recommended with mifepristone [6, 11]. Liver function testing was performed given rare cases of liver injury with long-term use of UPA for treatment of uterine fibroids [12].

Participants underwent a transvaginal ultrasound on day 3 to ensure pregnancy expulsion. Participants without complete expulsion were offered an additional dose of misoprostol, a surgical procedure, or expectant management. Participants who needed an additional dose of misoprostol or expectant management following their day 3 visit were scheduled to return on day 8 following initial treatment to confirm pregnancy expulsion. Liver function testing was repeated on day 14. Study participants were followed through 30 days from treatment to identify any adverse events or need for additional treatment, regardless of pregnancy expulsion at their day 3 follow up. All study participants received a phone call on day 30 to self-report any side effects, adverse events, or additional treatments, and to assess acceptability of the treatment. Side effects investigated included fatigue, headache, dizziness or lightheadedness, chills, nausea, diarrhea, vomiting, severe cramping, or fever; adverse events captured included excess bleeding requiring hospitalization and/or blood transfusion or pelvic infection requiring hospitalization and/or antibiotic administration; all based on side effects and adverse events reported on the mifepristone product label [11].

## Results

We recruited patients from May 2022 through April 2023. Eight patients expressed interest in the study, and three enrolled. Multiple modifications were made to increase recruitment, including weekly emails to physicians reading early pregnancy ultrasounds, monthly reminders to all OBGYN faculty and residents seeing pregnant patients, and revised recruitment flyers and pamphlets based on consultation with recruitment specialists at our institution.

The three participants enrolled had a median gestational age of 10 weeks (range 8–11 weeks) and were all non-Hispanic white, married, and with Bachelors’ degrees and private insurance. All adhered to the study protocol, completed all study visits, and had resolution of their pregnancy without the need for additional medication or surgical intervention. No elevation in liver function tests was noted at day 14.

One participant was admitted for observation following heavy vaginal bleeding but did not need a blood transfusion. This patient received 90 mg ulipristal acetate on study day 1 and took her misoprostol in the morning of day 2, per protocol. She had heavy bleeding and started feeling faint on the evening of day 2, which prompted her to call emergency medical services. The patient was noted to be hypotensive by emergency medical services and was given intravenous fluids and brought to the emergency department. Her hemoglobin dropped from a baseline of 11.7 g/dL to 10.1 g/dL, but the patient was noted to have minimal vaginal bleeding on exam. Her transvaginal ultrasound noted complete pregnancy expulsion. Patient was admitted for observation, but no additional intervention was needed.

The most common side effects at day 3 included fatigue (2/3 participants), dizziness (2/3 participants), nausea (1/3 participants) and cramping (1/3 participants). By day 30, only one out of three participants reported dizziness, chills, and severe cramping. Patients generally found the study intervention acceptable (Table 1).

**Table 1:** Acceptability of treatment by patient report by Likert Scale (1 = very unlikely, 2 = more unlikely, 3 = neutral, 4 = more likely, 5 = very likely) on study day 30 following UPA administration

Questions to Assess Acceptability	Likert Scale Response (median, range)
*Would you use these medications again if you had another miscarriage?*	3 (1, 5)
*Would you recommend these medications to a friend with a miscarriage?*	4 (3, 5)
*If it was possible to take the ulipristal acetate at home (versus in clinic)*,* would you be more or less likely to use these medications again?*	4 (3, 4)
*If it was possible to take the ulipristal acetate at a different time (versus in clinic)*,* would you be more or less likely to use these medications again?*	3 (3, 4)

## Conclusion

UPA 90 mg showed promise as an adjunct to misoprostol in treatment of EPL as evidenced by successful treatment in all three patients. However, unique barriers in our practice setting made this pilot study difficult to conduct, including the widespread availability of mifepristone for EPL at our institution. Many patients experiencing EPL expressed hesitation using an off-label medication during a vulnerable and emotional time.

Alternatives to mifepristone for early pregnancy loss care is important given the current REMS restrictions to mifepristone and the potential for legal challenges to the medication. Additionally, many women diagnosed with EPL are seen in non-OBGYN settings, such as the emergency department or primary care, where mifepristone prescribing restrictions often prevent patients from accessing appropriate evidence-based medical management. Indeed, in a large qualitative survey of primary care clinicians, two thirds of providers were unable to dispense mifepristone in their clinics for medication abortion or EPL care, with most citing logistical difficulties with the REMS restrictions and resistance from health center leadership as the rationale [13]. Logistical barriers to prescribing mifepristone for medication abortion have also been well documented among both OBGYN and non-OBGYN providers [14, 15]. Additionally, the availability of mifepristone in the United States may be more limited with legal and policy changes. Thus, studying alternatives to mifepristone is a key public health need currently.

Overall, UPA showed promise in our pilot study as an effective agent as evidenced by successful treatment in all three patients enrolled with resolution of EPL by three days following medication administration. In addition, the treatment was well tolerated with minimal side effects. While prolonged use of UPA has been associated with rare cases of liver injury, none of the patients in our study had increases in their liver function tests from baseline. Even with concerns for rare cases of liver with prolonged use of UPA for uterine fibroid treatment, UPA at emergency contraception dosing (30 mg) has always been identified as safe and appropriate to use [16]. Our data suggests that a single 90 mg dose of UPA may be similarly safe with no changes to liver function testing. However, with a small sample size it is challenging to make broad conclusions regarding this regimen.

The study was limited due to its small sample size despite multiple efforts to increase recruitment during the study period. Unique barriers in our practice setting made this pilot study difficult to conduct, including the widespread availability of mifepristone for EPL during the study period. Patients expressed hesitation about using a new and off-label study medication that only had a hypothetical benefit over the standard treatment (with mifepristone and misoprostol), particularly during a vulnerable and emotional time following diagnosis of EPL. The concept of potential future benefits of UPA if mifepristone became difficult to access was challenging for many patients to grasp. Those who did enroll cited altruistic reasons to further science and help patients in the future. Additionally, as our study team was only available during specific times for intake visits, some patients opted for medical management with mifepristone and misoprostol due to the desire to proceed with treatment at a time more convenient for them. Finally, while the 90 mg dose of UPA was chosen to most closely match mifepristone 200 mg based on endometrial histology, this higher dose also necessitated liver function testing, which was often burdensome on patients both at their baseline and two week follow up visits. The study of UPA in addition misoprostol for medication abortion studied doses of 60 mg and 90 mg UPA and found similar efficacy and safety profiles without requiring liver function testing for either dose [7]. Future studies could use 30 mg UPA, negating the need for liver function testing, which may make follow up more acceptable to patients.

As this was the first time UPA has been studied as an adjunct to misoprostol for this indication, we performed a single arm study. However, based on these preliminary results, a larger randomized non-inferiority trial of mifepristone versus UPA as adjuncts to misoprostol may be warranted to compare options for EPL treatment, particularly given the success of UPA plus misoprostol in medication abortion [7]. Alternatively, testing UPA plus misoprostol versus misoprostol alone may elucidate the exact effects of UPA on management of early pregnancy loss, and may be necessitated if mifepristone becomes unavailable. Other adjuncts to misoprostol, such as letrozole, have also been explored as alternatives to mifepristone, with non-inferior outcomes in outcomes of EPL [17]. Data on alternative options for treatment of EPL may improve access, allowing equitable and patient centered miscarriage care.

## Data Availability

The datasets used and analyzed during the current study are available from the corresponding author on reasonable request.

## Abbreviations

EPL: Early pregnancy loss
FDA: Food and Drug Administration
REMS: Risk Evaluation and Mitigation Strategy
UPA: Ulipristal acetate
